# CD30-targeted oncolytic viruses as novel therapeutic approach against classical Hodgkin lymphoma

**DOI:** 10.18632/oncotarget.24191

**Published:** 2018-01-12

**Authors:** Julia D.S. Hanauer, Benjamin Rengstl, Dina Kleinlützum, Johanna Reul, Anett Pfeiffer, Thorsten Friedel, Irene C. Schneider, Sebastian Newrzela, Martin-Leo Hansmann, Christian J. Buchholz, Alexander Muik

**Affiliations:** ^1^ Molecular Biotechnology and Gene Therapy, Paul-Ehrlich-Institut, Langen, Germany; ^2^ Dr. Senckenberg Institute of Pathology, Goethe-University Frankfurt, Frankfurt am Main, Germany; ^3^ Current address: BioNTech RNA Pharmaceuticals GmbH, 55131 Mainz, Germany; ^4^ Current address: BioNTech Cell and Gene Therapies GmbH, 55131 Mainz, Germany

**Keywords:** classical Hodgkin lymphoma, CD30, virotherapy, retargeting, VSV

## Abstract

Classical Hodgkin lymphoma (cHL) is a hematopoietic malignancy with a characteristic cellular composition. The tumor mass is made up of infiltrated lymphocytes and other cells of hematologic origin but only very few neoplastic cells that are mainly identified by the diagnostic marker CD30. While most patients with early stage cHL can be cured by standard therapy, treatment options for relapsed or refractory cHL are still not sufficient, although immunotherapy-based approaches for the treatment of cHL patients have gained ground in the last decade. Here, we suggest a novel therapeutic concept based on oncolytic viruses selectively destroying the CD30^+^-positive cHL tumor cells. Relying on a recently described CD30-specific scFv we have generated CD30-targeted measles virus (MV-CD30) and vesicular stomatitis virus (VSV-CD30). For VSV-CD30 the VSV glycoprotein G reading frame was replaced by those of the CD30-targeted MV glycoproteins. Both viruses were found to be highly selective for CD30-positive cells as demonstrated by infection of co-cultures of target and non-target cells as well as through blocking infection by soluble CD30. Notably, VSV-CD30 yielded much higher titers than MV-CD30 and resulted in a more rapid and efficient killing of cultivated cHL-derived cell lines. Mouse tumor models revealed that intratumorally, as well as systemically injected VSV-CD30, infected cHL xenografts and significantly slowed down tumor growth resulting in a substantially prolonged survival of tumor-bearing mice. Taken together, the data support further preclinical testing of VSV-CD30 as novel therapeutic agent for the treatment of cHL and other CD30^+^-positive malignancies.

## INTRODUCTION

Classical Hodgkin lymphoma (cHL) is a malignant disease of the hematopoietic system and occurs with an incidence of 3–4 cases per 100.000 persons per year [1]. cHL is characterized by a unique histological pattern, as tumor cells of cHL are composed of Hodgkin and Reed-Sternberg (HRS) cells representing a mononucleated or multinucleated subtype, respectively. The most important immunological markers on these HRS cells are CD30 and CD15. Importantly, cHL cells only account for < 1% of the tumor mass. The tumor cells are accompanied by a characteristic reactive infiltrate, mainly consisting of activated lymphocytes [2]. This implies that very few neoplastic cells recruit a great amount of lymphocytes and create a microenvironment favorable for tumor progression. Especially CD4^+^ T cells are involved in the so-called rosetting of tumor cells. Their presence correlates with poor progression-free and overall survival [3]. Current standard treatment for patients with early stage cHL includes multi-agent chemotherapy and localized radiotherapy [4]. Patients with chemosensitive relapse of cHL are treated by autologous hematopoietic stem cell transplantation (HSCT). This treatment results in long-term progression-free survival in approximately 50% of patients [5]. Therapeutic concepts resulting in a permanent antitumoral response in patients who failed autologous HSCT are therefore still needed. Recent concepts have started to focus on immunotherapy approaches. As targeting molecule, CD30 is in focus since it is minimally expressed on normal cells [6]. The most prominent example for a CD30-directed medicinal product is brentuximab vedotin, a CD30-specific monoclonal antibody coupled to a cytotoxic microtubule disrupting agent, which recently received marketing authorization [6]. Applied as salvage therapy for patients with relapsed cHL and with a 41% 5-year overall survival rate it has substantially improved the situation for these patients [7]. Further improvement can be expected from the PD1-specific immune checkpoint inhibitor nivolumab, which was approved in 2016 for the treatment of patients with cHL that have relapsed or progressed after HSCT and brentuximab vedotin treatment [5]. However, with a 65% objective response rate there is still need for improvement and alternative concepts.

Oncolytic viruses combine the selective destruction of tumor cells with the induction of an antitumoral immune response [8]. They have so far been developed for many cancer entities and reached marketing authorization for the treatment of melanoma with the herpesvirus-based product Imlygic [9]. For cHL, however, virotherapy has so far not been considered. The low frequency of tumor cells in the affected tissue, as compared to other tumor entities, makes the use of conventional oncolytic viruses challenging. Additional layers of tumor cell targeting may in the case of cHL be instrumental to establish an effective virotherapy strategy. Cell type specific infection at the level of cell entry has been established for some enveloped oncolytic viruses such as measles virus (MV) or herpesvirus [10, 11]. In case of MV, cell entry is mediated by the receptor attachment protein hemagglutinin (H) and the membrane fusion protein F. Usage of the natural receptors CD46, signaling lymphocyte activation molecule (SLAM), and nectin-4 for cell entry can be redirected to a cell surface receptor of choice by introducing point mutations in H and fusion to a targeting ligand with high affinity for the selected surface marker. By this means a series of measles viruses have been generated each using a particular type of tumor surface marker as receptor [12, 13]. All these viruses are highly selective for their target cells and replicate as efficiently as unmodified MV. Recently it was shown that vesicular stomatitis virus (VSV) can be receptor-targeted by replacing its glycoprotein G gene against the glycoproteins of MV. Compared to MV, it replicates faster and can be produced at higher titers [14, 15].

In the present work, we generate the first examples of CD30-targeted oncolytic viruses. We compare the oncolytic activities of MV and VSV on cHL tumor cell lines *in vitro* and *in vivo*. VSV-CD30 was found to be especially promising for further preclinical studies, since it was active also upon systemic administration and in some mice even in a disseminated tumor mouse model.

## RESULTS

### Generation of CD30-targeted oncolytic viruses

**Figure 1:**
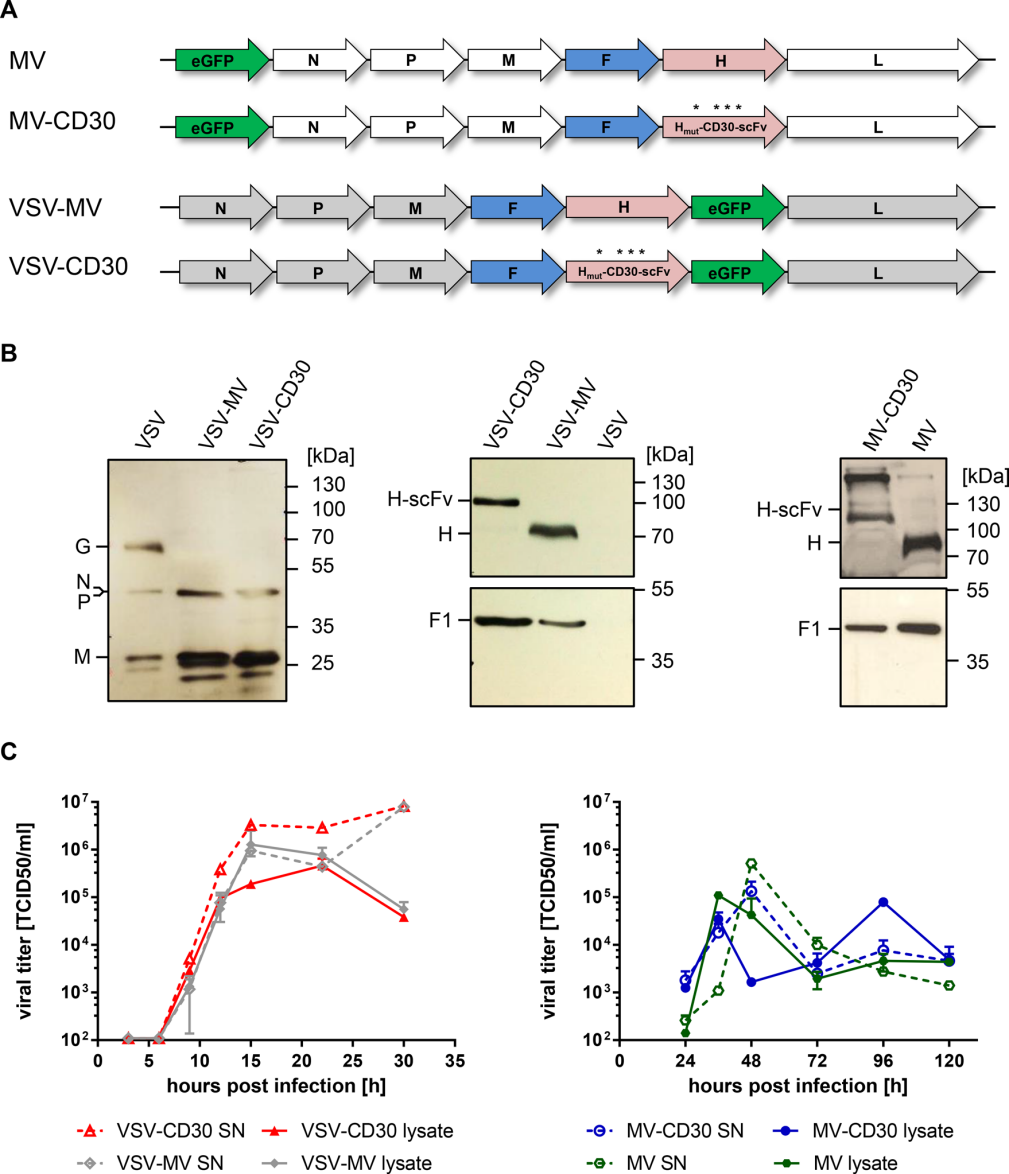
Generation of MV-CD30 and VSV-CD30 (**A**) Schematic genome organization of the applied oncolytic viruses. Asterisks indicate the mutated residues in H protein to achieve blinding for the natural MV receptors CD46 and SLAM. The coding sequence for the CD30-specific scFv together with a C-terminal hexa-His-tag (H6) is fused to H_mut_. In VSV-CD30 the glycoprotein (G) reading frame was replaced with those of MV-F and H_mut_-CD30scFv (**B**) Immunoblot of virus stocks using rabbit-α-VSV serum (left panel) or antibodies recognizing the cytoplasmic tail of MV-H (center and right panel top row) or MV-F (center and right panel bottom row). (**C**) Multi-step growth curves of CD30-targeted viruses and untargeted parental viruses on Vero-αHis cells of cell-associated (lysate) and supernatant (SN) virus after infection at a multiplicity of infection (MOI) of 0.3, respectively. Titers were determined as 50% tissue culture infective dose (TCID50). *n* = 2, error bars: mean ± SD.

With the aim of selectively destroying CD30^+^-positive cHL-cells, both, CD30-targeted measles virus (MV-CD30) and vesicular stomatitis virus (VSV-CD30), were generated. As CD30-specific binding domain the recently described scFv HRS3opt2#2 was used [16]. Its coding sequence was fused to that of the MV hemagglutinin (H) variant, which has been blinded for usage of the natural MV receptors CD46 and SLAM [17]. For MV-CD30 the unmodified MV-H gene was exchanged against the coding sequence of H_mut_-CD30scFv, whereas in case of VSV-CD30 the VSV glycoprotein G gene was replaced by the reading frames of the MV fusion protein (F) and H_mut_-CD30scFv (Figure 1A). After rescue of the oncolytic viruses (OVs), they were propagated on Vero-αHis cells, which are CD30-negative but display a Hexa-His-tag (H6)-specific antibody which can be used as entry receptor by both viruses due to a C-terminal H6 fused to H_mut_-CD30scFv. As a control MV-CD30, VSV-MV, VSV-CD30 containing an unmodified H protein (without H6) was rescued as well.

To verify the molecular composition of the rescued viruses, Western blot analysis was performed. VSV-CD30 and VSV-MV contained the MV protein F and H along with the VSV proteins N, P and M (Figure 1B). The VSV G protein was only detectable in stocks of VSV but not in the VSV-MV chimeric viruses. In correspondence to the fused scFv protein, the electrophoretic mobility of H_mut_-CD30scFv was reduced when compared to H. This was also the case for stocks of MV-CD30 which were analyzed along with MV stocks (Figure 1B). The incorporation of the CD30-scFv did not influence the replication of VSV-CD30 and MV-CD30. Replication kinetics of both viruses did not differ from those of their parental viruses (Figure 1C). Notably, VSV-CD30 and VSV-MV replicated faster and to higher titers than their MV-based counterparts.

### Receptor tropism of the CD30-targeted viruses

**Figure 2:**
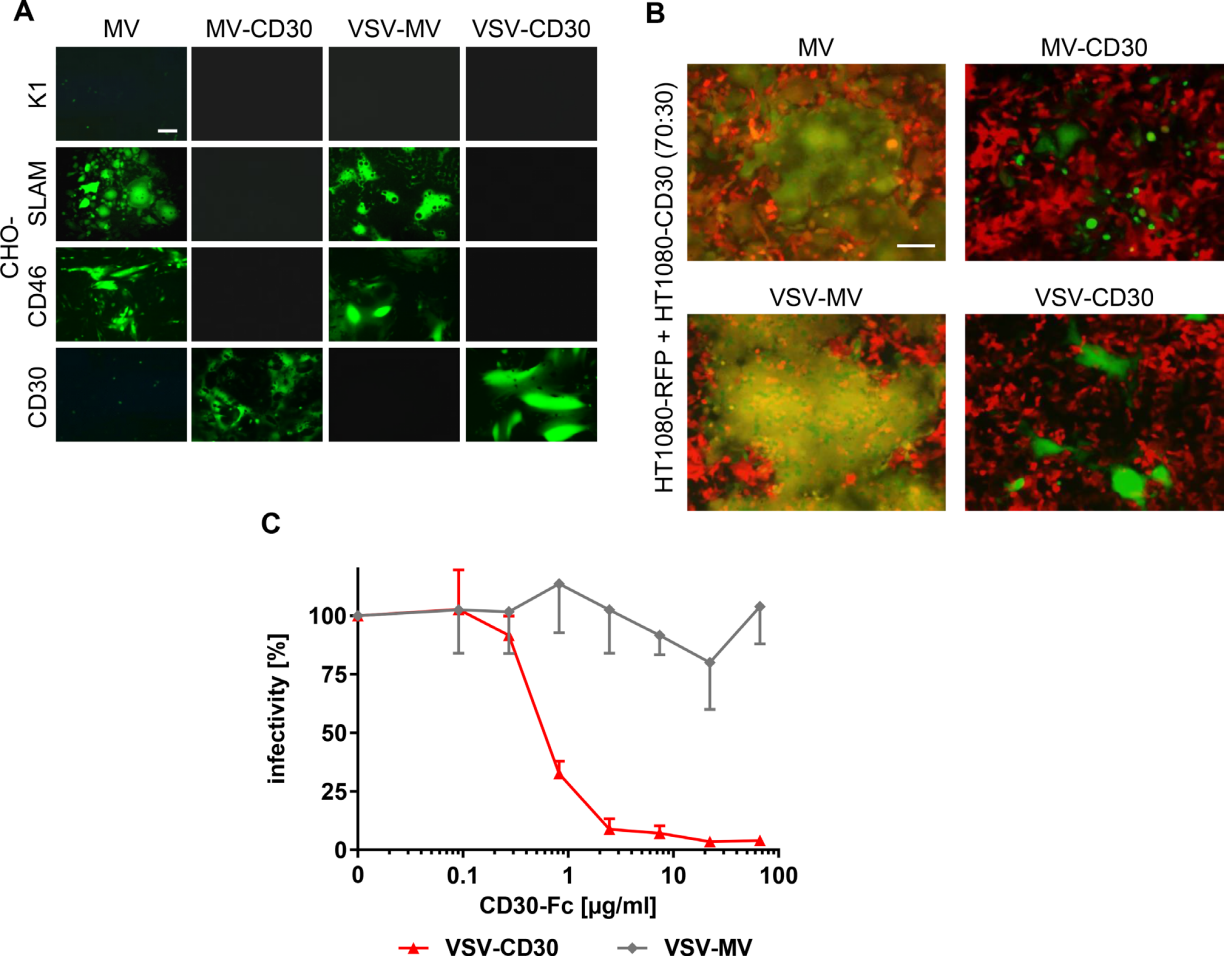
Receptor usage of MV-CD30 and VSV-CD30 (**A**) CHO-cells stably expressing SLAM, CD46 or CD30 were infected with CD30-targeted viruses or untargeted parental viruses at an MOI of 1 and analyzed by fluorescence microscopy 24 h (VSV-MV, VSV-CD30) or 72 h (MV, MV-CD30) post infection, respectively. Scale bar = 200 μm. (**B**) HT1080-RFP and HT1080-CD30 were co-cultured at a ratio of 70:30 and infected with CD30-targeted viruses or untargeted parental viruses at an MOI of 1 and analyzed by fluorescence microscopy 24 h (VSV-MV, VSV-CD30) or 72 h (MV, MV-CD30) post infection, respectively. An overlay of red and green fluorescence is shown. Scale bar = 200 μm. (C) VSV-MV and VSV-CD30 were pre-incubated for 30 min with increasing amounts of CD30-Fc protein. Subsequently, HT1080-CD30 cells were inoculated and the percentages of infected cells were determined 72 h later. Relative infectivities were calculated by normalizing the values to mock treated controls. *n* = 3, error bars: mean ± SD.

Usage of CD30 as entry receptor by the generated CD30-targeted viruses was analyzed on a panel of CHO cells stably expressing either the natural MV receptors CD46 or SLAM, or the target receptor CD30. Parental CHO-K1 cells that do not express any of the receptors were not infected, neither by the CD30-targeted viruses, nor their parental viruses (Figure 2A). While MV and VSV-MV infected CD46-positive and SLAM-positive cells, both CD30-targeted viruses exclusively infected CHO cells expressing CD30, thus indicating successful retargeting (Figure 2A). The selectivity of the CD30-targeted viruses for CD30-positive cells was further verified in a mixed cell culture composed of CD30-negative HT1080 and HT1080-CD30 cells. For better discrimination of the two cell types, CD30-negative HT1080-cells stably expressed the red fluorescent protein RFP (HT1080-RFP). Upon infection with the GFP encoding viruses these cells were expected to emit yellow fluorescence. Indeed, infection with MV or VSV-MV led to yellow fluorescence, mainly emitted from large syncytia that had formed between both cell types (Figure 2B). In sharp contrast, addition of MV-CD30 or VSV-CD30 to the co-culture resulted in green fluorescence emitting syncytia, while the red fluorescent cells did not turn yellow nor formed syncytia (Figure 2B). The data demonstrate that the CD30-targeted viruses selectively infect CD30-positive cells, even when these are in direct contact with CD30-negative cells. To finally prove that CD30 was used as entry receptor by VSV-CD30, we assessed competition of infection by soluble CD30. For this purpose, CD30-Fc, a fusion protein composed of the extracellular part of CD30 and the Fc-tag, was expressed and purified as described previously [16] and then pre-incubated with VSV-CD30 or VSV-MV before infection of HT1080-CD30 cells. The infectivity of VSV-CD30 decreased in a dose dependent manner, while that of VSV-MV remained unaffected (Figure 2C).

### Susceptibility of human classical Hodgkin lymphoma cell lines

**Figure 3:**
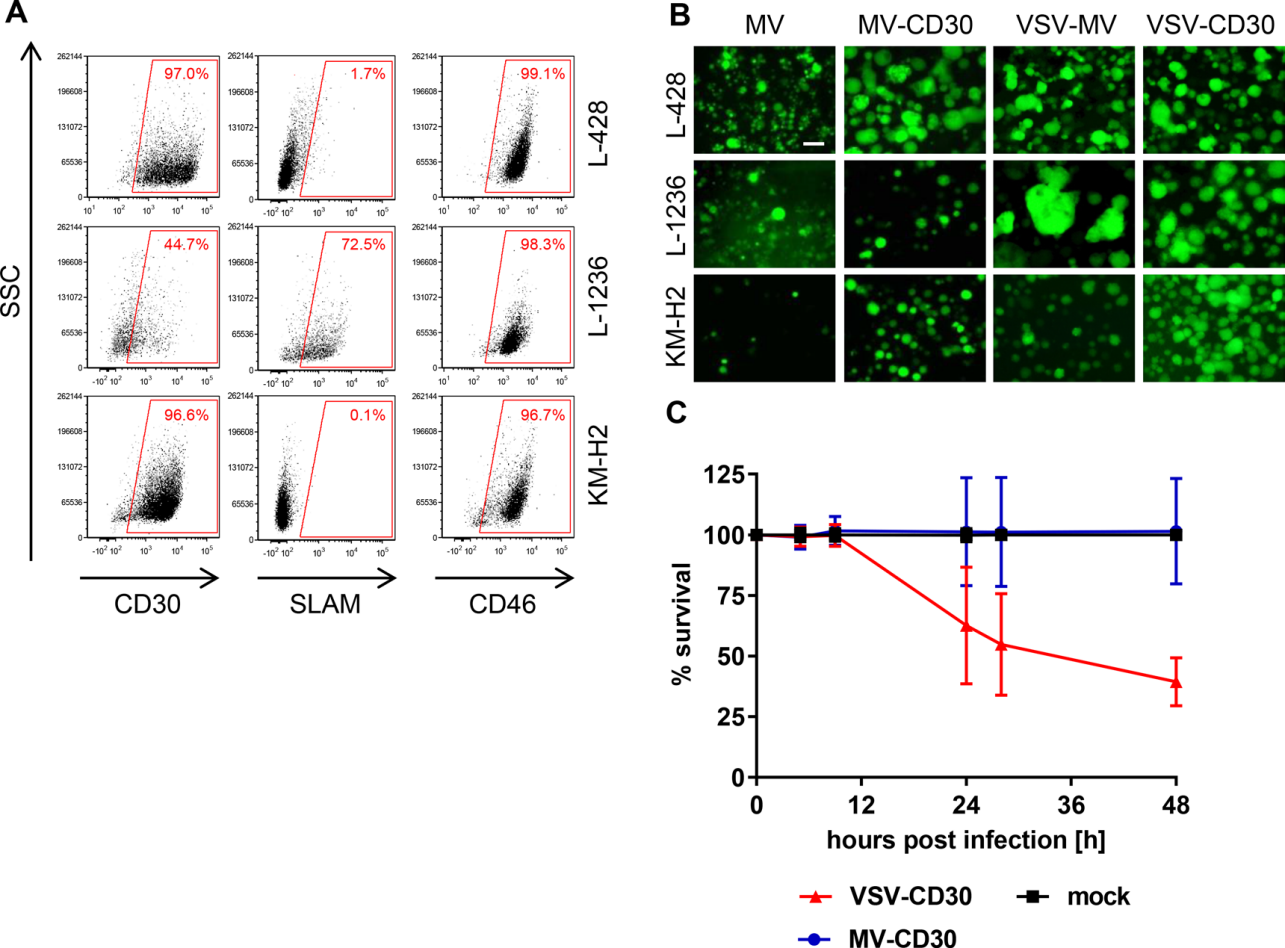
Infection of human classical Hodgkin lymphoma cell lines (**A**) FACS analysis of the human cHL cell lines L-428, L-1236 and KM-H2 for expression of CD30, SLAM and CD46. (**B**) The cHL cell lines L-428, L-1236 and KM-H2 were infected with the CD30-targeted or the untargeted parental viruses at an MOI of 1, respectively, and analyzed by fluorescence microscopy 24 h (VSV-MV, VSV-CD30) or 72 h (MV, MV-CD30) post infection. Scale bar = 200 μm. (**C**) Cell viability was determined using the RealTime-Glo MT Cell Viability Assay. Human KM-H2 cells were infected with MV-CD30 or VSV-CD30 an MOI of 1, respectively, and viability was determined until 48 h post infection. *n* = 3, Error bars: mean ± SD.

Due to the unavailability of primary HRS-cell cultures cHL patient-derived continuous cell lines were used. These covered two different subtypes of HL, nodular sclerosis (L-428) and mixed cellularity (KM-H2, L-1236) and represented long-standing adequate models for HRS cell physiology with tumorigenic potential [18–20]. Expression levels of the target receptor CD30 and the natural MV receptors CD46 and SLAM were determined by flow cytometry. Flow cytometry analysis confirmed that L-428 and KM-H2 cells expressed CD30 as well as CD46 in a similar high amount, but hardly any SLAM (Figure 3A). Less than half of the L-1236 cells expressed CD30, which were in contrast to the other cell lines not only CD46-positive but also SLAM-positive (Figure 3A). Nevertheless, both CD30-targeted viruses as well as their untargeted parental viruses readily infected all three cHL cell lines and induced the formation of large syncytia (Figure 3B). Finally, the cytotoxic effects exerted by MV-CD30 and VSV-CD30 on KM-H2 cells were determined. While within 48 h MV-CD30 did not yet kill cells, VSV-CD30 reduced the cell viability to 60% after 24 h and to 48% after 48 h (Figure 3C). Thus, VSV-CD30 was more potent in killing KM-H2 cells than MV-CD30 *in vitro*.

### Antitumoral activity of VSV-CD30 and MV-CD30 *in vivo*

**Figure 4:**
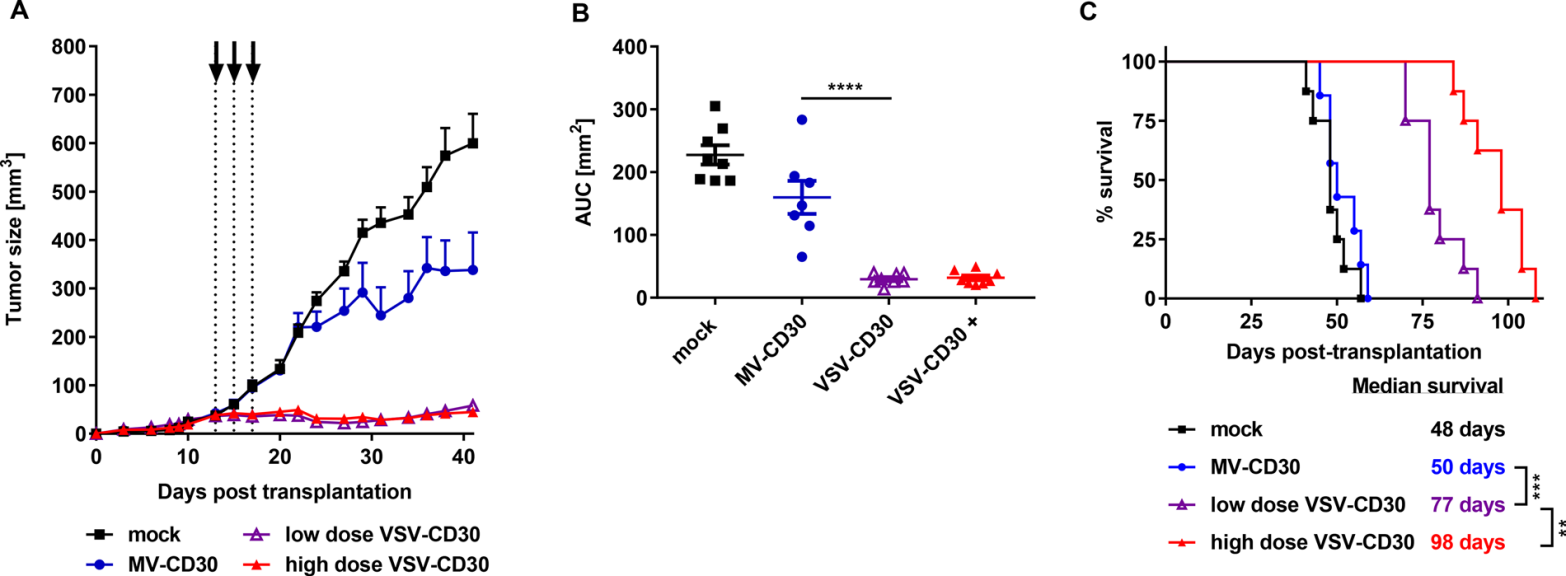
Oncolytic activity *in vivo* after intratumoral administration KM-H2 cells were implanted subcutaneously into NSG mice. 13 days post cell implantation, mice received three intratumoral injections over a period of five days (dotted lines) covering a total dose of 3 × 10^6^ TCID50 (MV-CD30, VSV-CD30 low dose) or 3 × 10^8^ TCID50 (VSV-CD30 high dose). (**A**) Tumor growth curves of MV-CD30 (blue circles), VSV-CD30 low dose (purple triangles), VSV-CD30 high dose (red triangles, +) or mock (black rectangles) treated mice. (**B**) Calculated area under the curve (AUC) values for tumor growth data shown in (A). One-way ANOVA test (Multiple comparisons), ^****^p < 0.0001. (**C**) Kaplan-Meier plot survival analysis. Logrank test (Bonferroni adjusted), ^***^p < 0.001, ^**^p < 0.01. Measles virus (MV)-CD30, *n* = 7; vesicular stomatitis virus (VSV)-CD30 low dose, *n* = 8; VSV-CD30 high dose, *n* = 8; mock, *n* = 8. Error bars: mean ± SEM.

Next, the antitumoral activities of VSV-CD30 and MV-CD30 were evaluated in the KM-H2 xenograft mouse model. KM-H2 cells were implanted subcutaneously into NSG mice. Once tumor had reached a volume of 50 mm^3^, three intratumoral (i.t.) administrations of in total 3 × 10^6^ TCID50 (MV-CD30 or VSV-CD30) or 3 × 10^8^ TCID50 (VSV-CD30) were performed within one week. Tumors of mice treated with MV-CD30 were only slightly reduced in growth compared to mock treated animals. In contrast, VSV-CD30 injected tumors were completely blocked in growth for several weeks (Figure 4A–4B). This held true not only for the high dose but also for the low dose, which was equivalent to that of MV-CD30. The oncolytic activity resulted in a significantly prolonged survival of tumor-bearing mice treated with VSV-CD30, which was further increased upon the high dose administration (Figure 4C).

**Figure 5:**
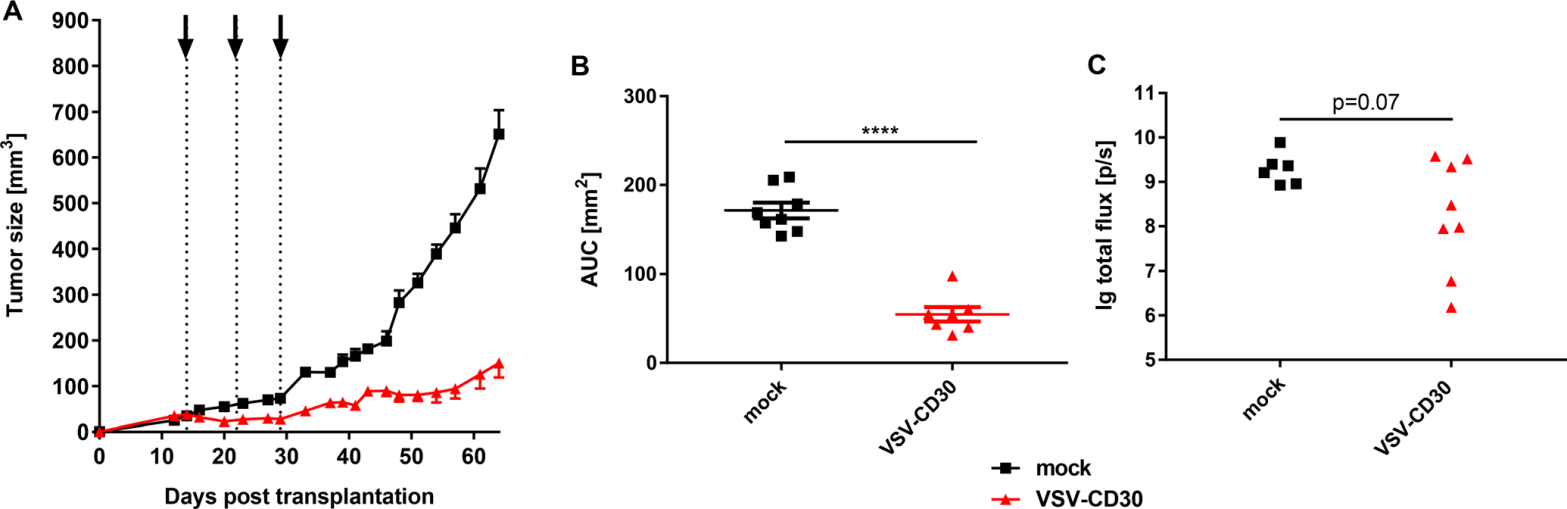
Oncolytic activity *in vivo* after systemic administration of VSV-CD30 (**A**) KM-H2 cells were implanted subcutaneously into NSG mice. 14 days post cell implantation, a total dose of 3 × 10^8^ TCID50 of VSV-CD30 split in three aliquots was systemically injected weekly (dotted lines). Tumor growth curves of VSV-CD30 (red triangles) or mock (black rectangles) treated mice are shown. (**B**) Calculated area under the curve (AUC) values for tumor growth data shown in (A). Unpaired *t* test, ^****^p < 0.0001. Vesicular stomatitis virus (VSV)-CD30, *n* = 7; mock, *n* = 8. Error bars: mean ± SEM. (**C**) KM-H2-luc cells were injected intravenously into NSG mice. Based on the luciferase signal intensity on day 14, mice received three intravenous injections of VSV-CD30 covering a total dose of 3 × 10^8^ TCID50 on day 18, 21 and 28 post cell administration. Quantified luciferase signals on day 46 are shown as logarithm of the total flux (p/sec). Unpaired *T* test, *p* = 0.07. VSV-CD30, *n* = 8; mock, *n* = 6.

Next, the oncolytic potential of VSV-CD30 was assessed after systemic application in the s.c. KM-H2 xenograft mouse model. For this purpose, the animals received three intravenous (i.v.) injections of in total 3 × 10^8^ TCID50 VSV-CD30 or OptiMEM as control within three weeks. Remarkably, also after systemic application VSV-CD30 controlled tumor growth for several weeks and thus equally well as after intratumoral administration (Figure 5A–5B). Quantification of the area under the curve (AUC) revealed that the difference in tumor growth between VSV-CD30 and mock treated was highly significant. On the day the last mock mouse had to be sacrificed all VSV-CD30 treated animals were still alive indicating a clear survival benefit. Spots of infected cells were detected throughout the tumor tissue; equally well in mice that had been injected with VSV-CD30 i.t. as in i.v. injected mice (Supplementary Figure 1). Notably, there were no signs for any side-effects including neurotoxic effects induced by the oncolytic viruses, neither after intratumoral nor after systemic administration.

Finally, we injected KM-H2-luc cells, which were labeled with the luciferase gene to monitor tumor growth, intravenously into NSG mice followed by VSV-CD30 injection. Luciferase signal intensity of KM-H2-luc cells was monitored and quantified over time for each treatment group (Supplementary Figure 2). At the last day of observation, in all mock-treated animals a high luciferase signal derived from the KM-H2-luc tumor burden was detected. In contrast, five out of eight VSV-CD30 treated animals exhibited considerably decreased luciferase activities (Figure 5C). This indicates that VSV-CD30 is oncolytically active also in a multifocal tumor model.

## DISCUSSION

Here, we proved the generation of oncolytic viruses using CD30 as entry receptor. CD30 is a diagnostic marker for several lymphomas including cHL where it is uniformly expressed in the HRS cells [21]. Belonging to the superfamily of tumor necrosis factor (TNF) receptors and as typical type-1 transmembrane protein, CD30 encompasses a large elongated extracellular part and a cytoplasmic domain that mediates signaling upon binding of the TNF family member CD30L. Expression of CD30 is mainly confined to pathological conditions, which include besides cHL also activated T lymphocytes as they occur during virus infection or autoimmune diseases [22]. Activated T cells could therefore form a potential target for VSV-CD30 or MV-CD30.

In healthy cells, including T lymphocytes, however, viral infection induces antiviral mechanisms of the innate immune system for example the expression of type-I interferons [23]. Tumor cells, in contrast, accumulate defects in the innate immune system to escape the immune response. These defects render them sensitive towards infection especially with RNA viruses [24]. Indeed, primary human T cells turned out to be completely protected from infection with VSV-CD30 or VSV-MV, despite the presence of CD30 on the cell surface and several days of cell cultivation after incubation with high MOI (Supplementary Figure 3). In contrast, some infected cells and syncytia were detectable with MV and to a minor extent also with MV-CD30 (Supplementary Figure 3). Thus, CD30 targeting does not extend the tropism of VSV or MV to T lymphocytes. This is well in line with the previous observation that targeting these viruses to CD133 does not lead to productive infection of hematopoietic stem cells, most likely due to the high sensitivity against interferon [15]. Taking in addition the proven safety of high dose systemic administration of MV in multiple myeloma patients into account [25] we expect that targeting CD30 results in safe products exerting, if at all, only subtle side-effects.

Our approach of CD30-targeting relied on engineered MV envelope glycoproteins displaying the stability-engineered CD30-specific scFv HRS3opt2#2. This scFv was proven to be highly stable and specific in mediating gene delivery by lentiviral vectors pseudotyped with these MV glycoproteins [26]. By replacing the envelope genes in the genomes of MV and VSV with those encoding the CD30-targeted glycoproteins, MV-CD30 and VSV-CD30 were generated. Both viruses exhibited a restricted infection and efficient spreading within CD30^+^-positive cHL cells. When comparing their oncolytic activities VSV-CD30 turned out to be more efficient than MV-CD30. This is in line with previously published data on chimeric VSV-MV viruses targeted to Her2/neu or CD133 [15, 27]. VSV-CD30 thus is now the third example of a receptor-targeted VSV. Although there is an obvious tendency for VSV to be oncolytically more effective than MV this cannot be generalized and has to be evaluated for each type of tumor separately. Especially the strong sensitivity towards interferon might be disadvantageous for VSV on certain tumor types as recently shown for VSV-CD133 and glioma tumor spheres, which turned out to be more susceptible to the MV counterpart MV-CD133 [15].

For the three HL cell lines used in this study, there were no hints for any impairment of the oncolytic activity by interferons detectable suggesting that cHL may be well compatible with the VSV-mediated oncolytic activity. Especially *in vivo*, where tumor cell lines often become responsive to interferons [28], VSV-CD30 was highly active and prevented outgrowth of the subcutaneously injected cHL cells. Notably, this was the case not only after local intratumoral but also after systemic intravenous injection, again underscoring the high activity of VSV-CD30. Primary HRS cells are basically not recoverable from single cell suspensions of HL-affected lymph nodes or other biopsy specimen of HL patients. Accordingly, only few HL cell lines were established during the last decades. The HL cell lines assessed in our study were frequently used for research purpose in important studies [18, 19]. They display three independent patient cases, as they were derived from pleural effusions (KM-H2, L-428) or peripheral blood (L-1236), and cover two different subtypes of HL, namely nodular sclerosis (L-428) and mixed cellularity (KM-H2, L-1236). A preclinical model reflecting the low abundance of tumor cells within the affected tissue is therefore so far not available [29, 30]. Hence, we have assessed VSV-CD30 in a mouse model of disseminated growing cHL tumor cells and found a substantial reduction in tumor growth at least for some of the mice. This illustrates nicely the power of CD30 targeting and suggests that VSV-CD30 may also effectively access and kill cHL tumor cells in patients and thus become a novel therapeutic option for cHL.

An interesting property of cHL that was recently discovered refers to the presence of CD30^+^-positive extracellular vesicles (CD30-EVs) shed from cHL tumor cells [31]. Interestingly, the authors found that CD30-EVs positively contribute to the clinical efficacy of brentuximab vedotin. They demonstrated that CD30-EVs migrate to CD30-negative but CD30 ligand (CD30L)-positive bystander cells in the tumor environment, mainly consisting of mast cells and eosinophils. Brentuximab vedotin binds to CD30-EVs, is then carried to the bystander cells where it becomes internalized and cytotoxically active. Since the CD30-targeted viruses described here bind to CD30 similarly efficient and selective as antibodies, it can be expected that it will be transported via CD30-EVs as well. If this leads to infection and killing of the bystander cells in the tumor microenvironment will be an interesting issue to be further investigated.

Besides cHL also other types of cancer such as anaplastic large cell lymphoma (ALCL), cutaneous CD30-positive lymphoproliferative disorders, lymphomatoid papulomatosis, diffuse large B cell lymphoma or adult T cell leukemia can be CD30 positive [6, 21]. Beyond that, also solid tumors especially mesothelioma and germ cell tumors can express CD30 [32]. The data we present here for the oncolytic activity of VSV-CD30 warrant further testing of this virus not only for applications in cHL, but also in these other CD30-positive disorders. Before clinical trials can be envisaged preclinical testing of VSV-CD30 will have to be extended assessing the oncolytic activity on cHL patient biopsy material and to toxicity testing. The latter will have to focus especially on neurotoxicity which is a concern with VSV-derived oncolytic viruses [33]. Envelope modification as done here, however, has often resulted in attenuation of VSV [34]. Upon completion, VSV-CD30 will add to a growing list of CD30-targeted therapeutics that include besides antibodies also CD30-specific AAV vectors [35] and CAR T cells [30, 36]. Combining VSV-CD30 with these or other cancer therapeutics such as checkpoint inhibitors will be another future option to be explored.

## MATERIALS AND METHODS

### Cells

BHK-21 (ATCC CCL-10), HEK 293-T (ATCC CRL-11268) and CHO-K1 (ATCC CCL-61) cells were cultured in DMEM (Sigma-Aldrich, Germany) supplemented with 10% FCS (Biochrom, Germany) and 2 mM L-glutamine (Sigma-Aldrich, Germany). Generation and cultivation of Vero-αHis [17], CHO-CD46 [37] and CHO-SLAM [38] has been described earlier. CHO-CD30 were derived from CHO-K1 cells (ATCC CCL-61) by stable expression of CD30 which was achieved upon selection with 10 μg/ml puromycin. HT1080-CD30 [16] and HT1080-RFP cells [39] have been described. The human cHL cell lines KM-H2, L-428 and L-1236 were obtained from the German Collection of Microorganisms and Cell Cultures, Germany and cultured in RPMI 1640 (Biowest, France) supplemented with 10% FCS and 2 mM L-glutamine. KM-H2-luc cells were derived from KM-H2 cells by stable expression of the luciferase gene which was achieved upon selection with 10 μg/ml puromycin.

### Cloning and rescue of MV-CD30 and VSV-CD30

As targeting ligand for CD30 we used the stability optimized scFv HRS3opt2#2 [16]. For cloning of the genome plasmid of MV-CD30 the sequence for retargeted H was cut out of the expression plasmid (pCG-H_mut_-scFvCD30opt2.2-6His) [16] using PacI/SpeI restriction sites. PacI/SpeI flanking insert was transferred into the CMV-promotor driven MV_NSe_ genome plasmid p(+)PolII-MV_NSe_-GFP(N) to yield p(+)PolII-MV_NSe_-GFP(N)-αCD30opt2.2 (pMV-CD30). For rescue of MV-CD30 the PolII rescue system [40] was used.

For cloning of the genome plasmid of VSV-CD30 the sequence for retargeted H was cut out of the expression plasmid (pCG-H_mut_-scFvCD30opt2.2-6His) using SfiI/NotI restriction sites. The backbone plasmid for VSV-MV chimera (pMC11-VSVFH-eGFP) was described earlier where the VSV-G glycoprotein (1.6 kb) at position 4 of the full-length VSV genome was replaced by the MV-F (1.8 kb) and MV-H (2 kb) at positions 4 and 5 [14]. SfiI/NotI flanking insert was transferred into the T7-promotor driven VSV-MV genome plasmid pMC11-VSVFH-eGFP to yield pMC11-VSVFH-αCD30opt2.2 (pVSV-CD30).

VSV-CD30 was rescued as described previously for VSV-CD133, essentially by making use of the helper plasmids coding for VSV-N, -P and –L, BHK-21 cells, a complementing VSV-G encoding plasmid and the T7 RNA polymerase provided by a modified vaccinia virus Ankara (MVA-T7-Pol) [15]. The rescued chimeric virus was purified by single syncytia isolation. All viruses were grown and titrated on Vero-αHis cells to calculate the 50% tissue culture infective dose (TCID50/ml).

### Virus growth kinetics

2 × 10^5^ Vero-αHis cells were seeded in a 12-well plate. Cells were infected with an MOI of 0.03. Samples were harvested every 24 h. For this purpose, virus was collected from the supernatant by centrifugation and stored at –80°C for further analysis. For harvesting cell-associated virus, adherent cells were scraped into 1 ml OptiMEM (Thermo Fisher Scientific, USA) and lysed by freeze-thawing in liquid nitrogen. Cell debris was removed by centrifugation. Resulting cell-associated virus containing medium was stored at –80°C. Titers were determined as TCID50/ml on Vero-αHis cells.

### Immunoblotting

Immunoblotting was performed as described previously [15]. Membranes were incubated with rabbit sera recognizing MV-F (Abcam, Great Britain), MV-H (Abcam, Great Britain) for detection of MV-CD30 and MV, the cytoplasmic tail of MV-H [39] for detection of VSV-CD30, VSV-MV, and VSV, or rabbit-α-VSV serum as described [41].

### Flow cytometry analysis

Expression of human CD30 was detected by a phycoerythrin (PE)-labeled mouse CD30 antibody (clone: Ki-2, Miltenyi Biotech, Germany). Human CD46 was detected by a fluorescein isothiocyanate (FITC)-labeled mouse CD46 antibody (clone: MEM-258, BioLegend, USA). Human CD150 (SLAM) was detected by a PE-labeled mouse CD150 (SLAM) antibody (clone: A12 (7D4), BioLegend, USA). Viability of cells was analyzed using the LIVE/DEAD Fixable Violet Dead Cell Stain Kit (Molecular Probes, USA). Flow cytometry analysis was performed using the MACSQuant Analyzer 10 (Miltenyi Biotec, Germany) and data were analyzed using FCS Express version 4.

### Cytotoxicity assay

1 × 10^5^ KM-H2 cells were seeded in a 96-well and infected with VSV-CD30 or MV-CD30 at an MOI of 1. Cell viability was analyzed using the RealTime-Glo MT Cell Viability Assay (Promega, Germany) according to the manufacturer’s protocol.

### Immunofluorescence staining of cryo sections

Cryo-sections of tumor tissue were performed as described previously [15]. For immunofluorescence staining against CD31 slices were incubated with the rat anti-GFP antibody (Dianova, Germany) overnight at 4°C, followed by incubation with the donkey anti-rat Alexa647-coupled secondary antibody (Jackson ImmunoResearch, USA).

### Animal experiments

All animal experiments were carried out in compliance with the regulations of the German animal protection law. To analyze the antitumoral effect of oncolytic viruses in the s.c. HL xenograft model, 1 × 10^7^ KM-H2 cells in 50 μl PBS were mixed with 50 μl Matrigel Basement Membrane Matrix (Corning, USA) and implanted into the flank of 6–8 weeks old female NSG mice (Charles River, Germany). Tumor growth was monitored regularly using a digital caliper. When tumors became palpable (at an average size of 50 mm^3^), mice were randomized into groups. They received in total three intratumoral injections of oncolytic virus. MV-CD30 (1 × 10^6^ TCID50 per injection) or VSV-CD30 (low dose: 1 × 10^6^ TCID50, high dose: 1 × 10^8^ TCID50 per injection) were administered in 50 μl OptiMEM every second day. Mock control animals received intratumoral injections of 50 μl OptiMEM (Thermo Fisher Scientific, USA). For systemic application, 1 × 10^8^ TCID50 VSV-CD30 in 200 μl OptiMEM or OptiMEM only as mock control were injected into the tail vein. In total, mice received three injections, with one injection per week. Differences in tumor growth were afterwards quantified via determination of area under the curve (AUC) [42].

For the multifocal tumor model, 2 × 10^6^ KM-H2-luc cells stably expressing the luciferase gene, were injected in 200 μl PBS intravenously via the tail vein into 6–8 weeks old female NSG mice. To follow up tumor progression, luciferase signals were detected by *in vivo* Imaging (IVIS Spectrum; Perkin Elmer, Germany) after intraperitoneal injection of 150 μg D-luciferin (Perkin Elmer, Germany) per gram body weight. Imaging data were obtained 10 min after luciferin injection. 14 days post cell administration animals were separated into treatment groups according to the mean luciferase signal intensity calculated via the Living Image Software (Caliper Life Sciences). Mice received three intravenous injections via the tail vein of VSV-CD30 (1 × 10^8^ TCID50 per dose) in 200 μl or PBS as mock control within 10 days. Mice were euthanized when the tumor had reached a size of > 800 mm^3^ or when more than 20% of their body weight was lost.

## SUPPLEMENTARY MATERIALS FIGURES


