# Supplementary material for: CD30-targeted oncolytic viruses as novel therapeutic approach against classical Hodgkin lymphoma
Source: Oncotarget. 2018 Jan 12;9(16):12971–81. doi: 10.18632/oncotarget.24191 (PMC5849188; doi:10.18632/oncotarget.24191)
Supplement: Supplementary file 1 [file oncotarget-09-12971-s001.pdf]

CD30-targeted oncolytic viruses as novel therapeutic approach against classical Hodgkin lymphoma

SUPPLEMENTARY MATERIALS

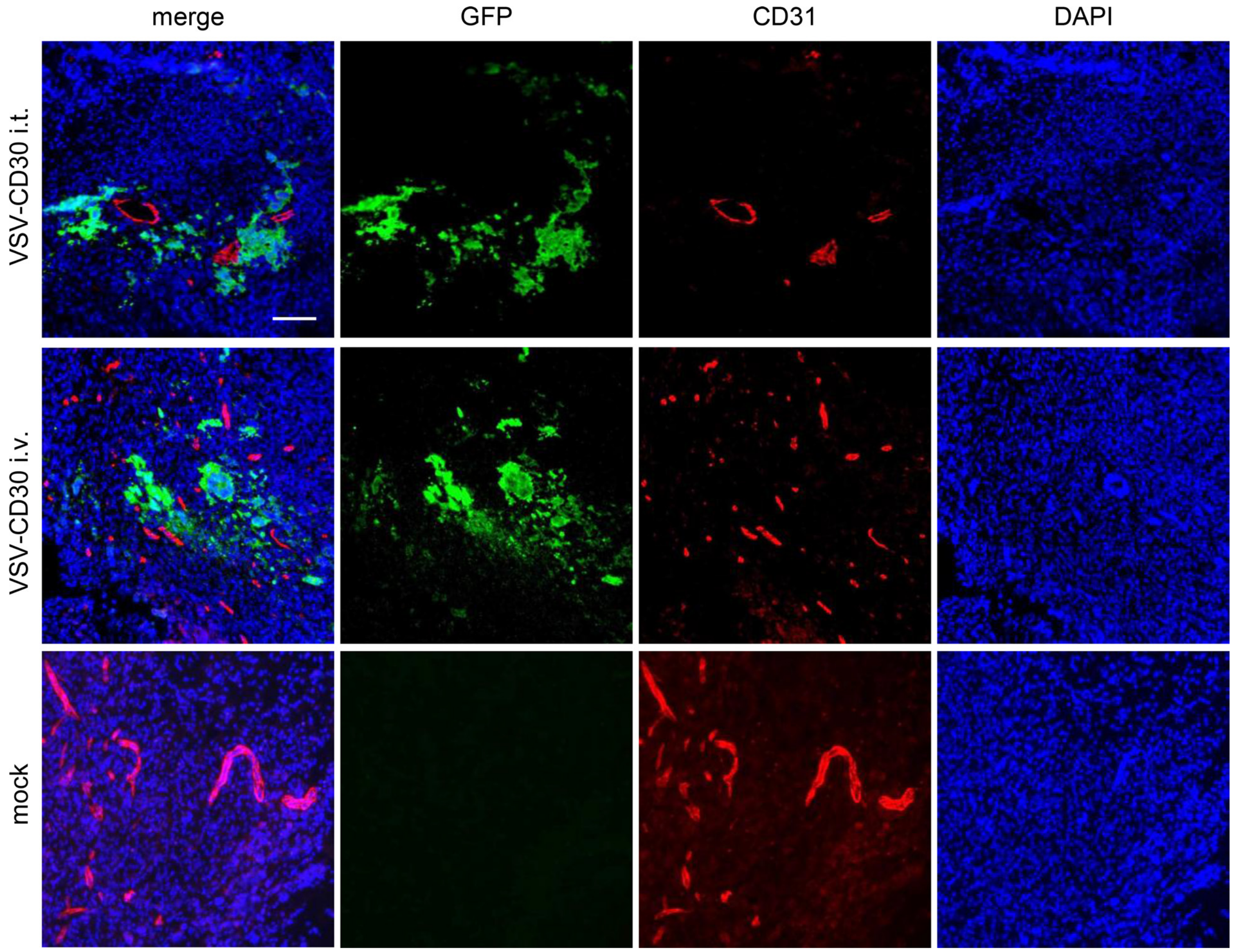

Supplementary Figure 1: Detection of VSV-CD30 in HL xenografts. Subcutaneously implanted KM-H2 tumors were treated with 1 x 10^6 TCID50 of VSV-CD30 intratumorally (top row), 1 x 10^8 TCID50 VSV-CD30 intravenously (middle row) or OptiMEM as mock control (bottom row). 14 days post treatment tumors were removed, cut in sections and GFP positive cells were detected by immunofluorescence staining using a polyclonal GFP antibody. Murine blood vessels were detected by immunofluorescence staining using a polyclonal CD31 antibody. One representative section out of 10 GFP positive sections is shown. Scale bar = 200 µm.

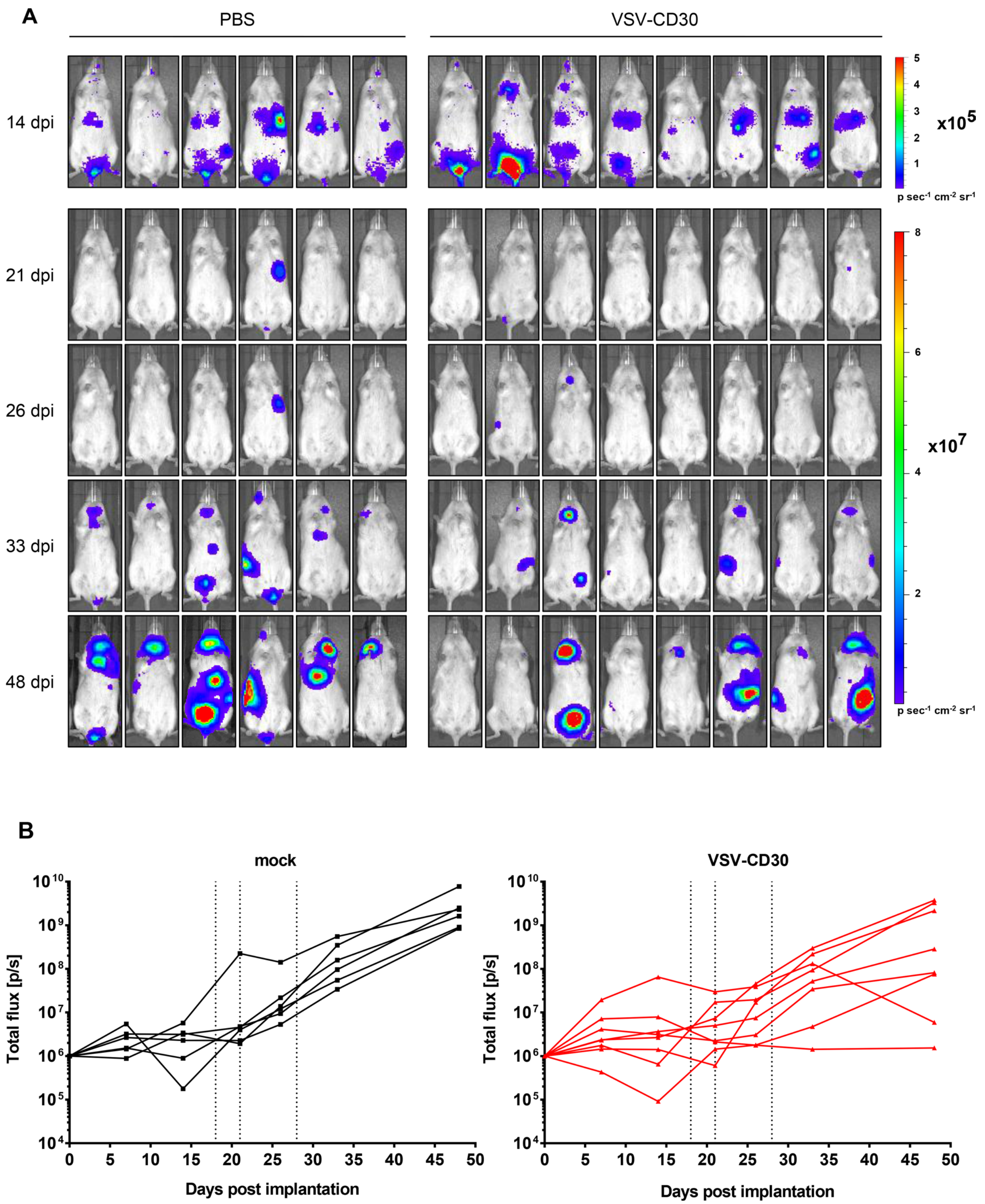

Supplementary Figure 2: Luciferase imaging raw data for disseminated tumor mouse model. Raw data for the analysis shown in Figure 5C. (**A**) Ventral view of all mice included in the experiment recorded at the indicated time points after tumor cell implantation. (**B**) Total luciferase activities over time for each individual mouse shown as spider plots. Dotted lines indicate the time points of mock injection (left diagram) or VSV-CD30 (right diagram), respectively.

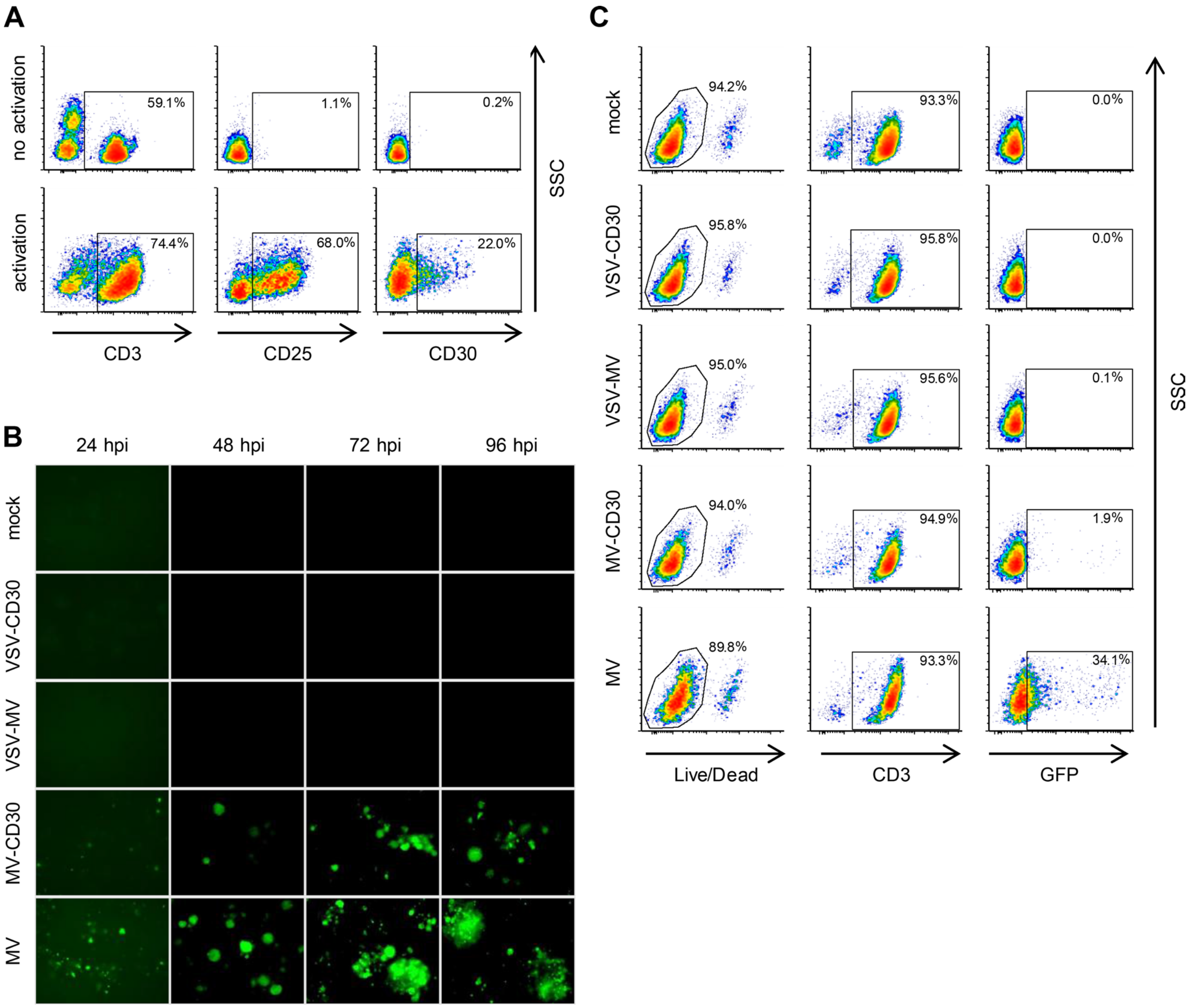

Supplementary Figure 3: Infection of primary human T lymphocytes. Human peripheral blood mononuclear cells (PBMC) were isolated from buffy coats and used for CD30 detection (**A**) or infection experiments (**B**, **C**). (**A**) FACS analysis of PBMC cultivated in absence (upper panel) or presence of the activating agents anti-CD3, anti-CD28 and IL-2 (lower panel) for the expression of CD3 (antibody clone: UCHT1), CD25 as activation marker (antibody clone: BC96) and CD30. (**B**) Activated PBMC were infected with VSV-CD30, VSV-MV, MV-CD30 or MV at an MOI of 0.1, respectively. GFP expression was monitored by fluorescence microscopy at the indicated time points post infection. (C) FACS analysis of activated PBMC 72 h after infection with VSV-CD30, VSV-MV, MV-CD30 or MV (MOI 1) for living cells (left panel), CD3 (center panel) and GFP (right panel).
